# Does number of rods matter? 4-, 5-, and 6-rods across a lumbar pedicle subtraction osteotomy: a finite element analysis

**DOI:** 10.1007/s43390-022-00627-0

**Published:** 2022-12-09

**Authors:** Niloufar Shekouhi, Ardalan S. Vosoughi, Vijay K. Goel, Alekos A. Theologis

**Affiliations:** 1 Engineering Center for Orthopedic Research Excellence (E-CORE), Departments of Bioengineering and Orthopaedic Surgery, University of Toledo, Toledo, OH USA; 2 Department of Orthopaedic Surgery, University of California-San Francisco (UCSF), 500 Parnassus Ave, MUW 3Rd Floor, San Francisco, CA 94143 USA

**Keywords:** Finite element analysis, Biomechanics, Pedicle subtraction osteotomy, Multi-rod constructs, Rod fracture, Pseudarthrosis

## Abstract

**Purpose:**

To assess biomechanics of a lumbar PSO stabilized with different multi-rod constructs (4-, 5-, 6-rods) using satellite and accessory rods.

**Methods:**

A validated spinopelvic finite element model with a L3 PSO was used to evaluate the following constructs: 2 primary rods T10-pelvis (“Control”), two satellite rods (4-rod), two satellite rods + one accessory rod (5-rod), or two satellite rods + two accessory rods (6-rod). Data recorded included: ROM T10-S1 and L2-L4, von Mises stresses on primary, satellite, and accessory rods, factor of safety yield stress, and force across the PSO surfaces. Percent differences relative to Control were calculated.

**Results:**

Compared to Control, 4-rods increased PSO flexion and extension. Lower PSO ROMs were observed for 5- and 6-rods compared to 4-rods. However, 4-rod (348.6 N) and 5-rod (343.2 N) showed higher PSO forces than 2-rods (336 N) and 6-rods had lower PSO forces (324.2 N). 5- and 6-rods led to the lowest rod von Mises stresses across the PSO. 6-rod had the maximum factor of safety on the primary rods.

**Conclusions:**

In this finite element analysis, 4-rods reduced stresses on primary rods across a lumbar PSO. Although increased rigidity afforded by 5- and 6-rods decreased rod stresses, it resulted in less load transfer to the anterior vertebral column (particularly for 6-rod), which may not be favorable for the healing of the anterior column. A balance between the construct’s rigidity and anterior load sharing is essential.

**Supplementary Information:**

The online version contains supplementary material available at 10.1007/s43390-022-00627-0.

## Introduction

Pedicle subtraction osteotomy (PSO) is a surgical technique to restore sagittal balance in patients with regional spinopelvic malalignment and global sagittal and/or coronal imbalance. The inherent degree of bone removal required in lumbar PSOs (i.e., posterior elements, bilateral pedicles, adjacent facets) creates an environment that is highly prone to pseudarthrosis and rod failure [1–5]. To reduce incidence of non-unions associated with PSOs, multi-rod constructs are now frequently used [6–14]. Four-rod constructs have traditionally been the most common rod configuration; however, more recently, there has been a trend toward utilization of additional rods (i.e., 5- and 6-rods) spanning lumbar PSOs with the goal to further reduce the risk of pseudarthrosis and rod breakage.

Multi-rod configurations can be created using “satellite” rods (not connected to primary rod) and/or “accessory” rods (connected to primary rod) [15]. While biomechanical properties of 4-rods (two primary rods + two satellite rods or two accessory rods) [10, 11, 13, 16–19], have previously been reported, there is limited understanding of the relative biomechanical behavior of “super” multi-rod constructs (5-, 6-rods) spanning a lumbar PSO. As such, the aim of this study is to evaluate the biomechanical characteristics of increasing number of rods (4-, 5-, 6-rod) across a lumbar PSO.

## Methods

**Fig. 1:**
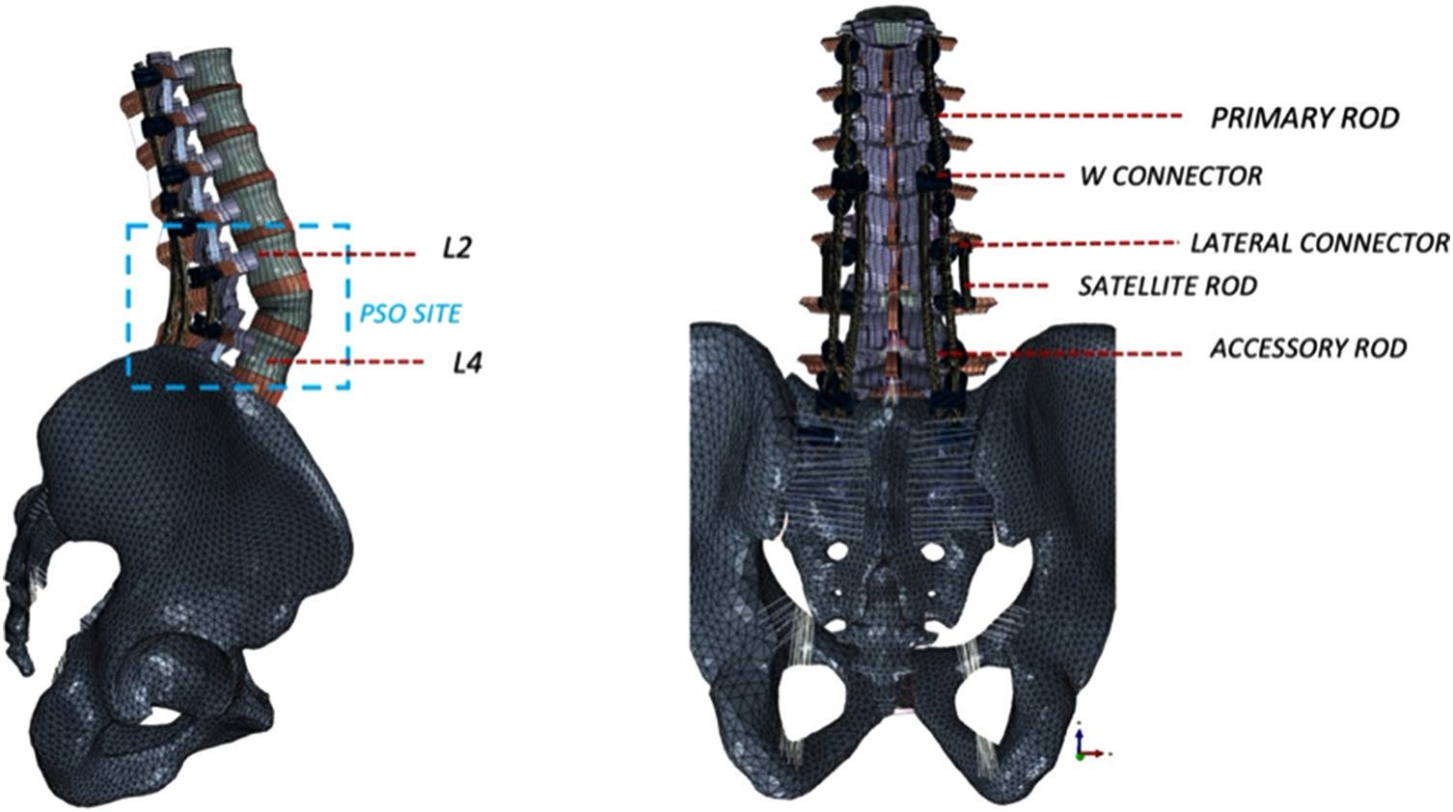
Sagittal (left) and anteroposterior (right) views of the modeled L3 pedicle subtraction osteotomy stabilized with a 5-rod construct. Posterior instrumentation includes pedicle screws, offset/lateral connectors, rod–rod connectors (open up-open up; “W”) as well as the rods (primary, satellite, and accessory)

A previously validated three-dimensional osseo-ligamentous spinopelvic finite element model (T10-pelvis) with a 30° PSO at L3 was used [16] (Fig. 1). The initial intact model of the ligamentous spine was reconstructed from computed tomography (CT) scans of a human spine using MIMICS (Materialize Inc., Leuven, Belgium) software. The IAFE-MESH (University of Iowa, Iowa) and HyperMesh (Altair Engineering, Michigan, USA) software were used to create hexahedral elements (C3D8) of the vertebrae and tetrahedral elements (C3D4) of the pelvis. The meshed components were assembled in the Abaqus 6.14 (DassaultSystemes, Simulia Inc., Providence, RI, USA) software. The spinal and sacroiliac ligaments were modeled using truss elements. In the vertebral body, a layer of 0.5 mm cortical bone was simulated to surround the cancellous bone.

The intervertebral disks were composed of annulus fibrosis and nucleus pulposus. The annulus fibrosis was simulated using a solid ground substance (C3D8 elements) that was reinforced with rebar elements (embedded into the ground matrix with ± 30° angles). The nucleus pulposus was modeled using C3D8 elements with hyper-elastic Mooney–Rivlin formulation. The sacroiliac joint was modeled using soft contact with exponential behavior. The material properties were assigned to each component based on the literature (Table 1) [16].

**Table 1:** Material properties used in model development

Components	Element formulation	Young’s modulus (MPa)/Poisson’s ratio
Vertebral cortical bone	Isotropic, elastic hex elements (C3D8)	12,000/0.3
Vertebral cancellous bone	Isotropic, elastic hex elements (C3D8)	100/0.2
Pelvic cortical bone	Isotropic, elastic hex elements (C3D8)	17,000/0.3
Pelvic cancellous bone	Isotropic, elastic hex elements (C3D8)	10/0.2
Annulus (ground)	Neo-Hookean, hex elements (C3D8)	C10 = 0.348, D1 = 0.3
Annulus (fiber)	Rebar	357−550
Nucleus	Mooney–Rivlin hex elements (C3D8H)	C1 = 0.12, C2 = 0.03, D1 = 0.0005
Apophyseal joints	Nonlinear soft contact, GAPUNI elements	–
Sacroiliac joints	Nonlinear soft contact	–
Ligaments	Hypo-elastic, tension only, Truss elements (T3D2)	Nonlinear stress−strain curves
Ti6Al4V pedicle screws	Isotropic, tetrahedron elements (C3D4)	11,500/0.3
CoCr rods	Isotropic, tetrahedron elements (C3D4)	241,000/0.3

Parameters derived from prior literature [16]

The L3 PSO was previously performed and validated [16] (Fig. 1). The anterior section was tied, while at the posterior, a surface-to-surface interaction (friction = 0.46) was defined between the two resected segments [16].

**Fig. 2:**
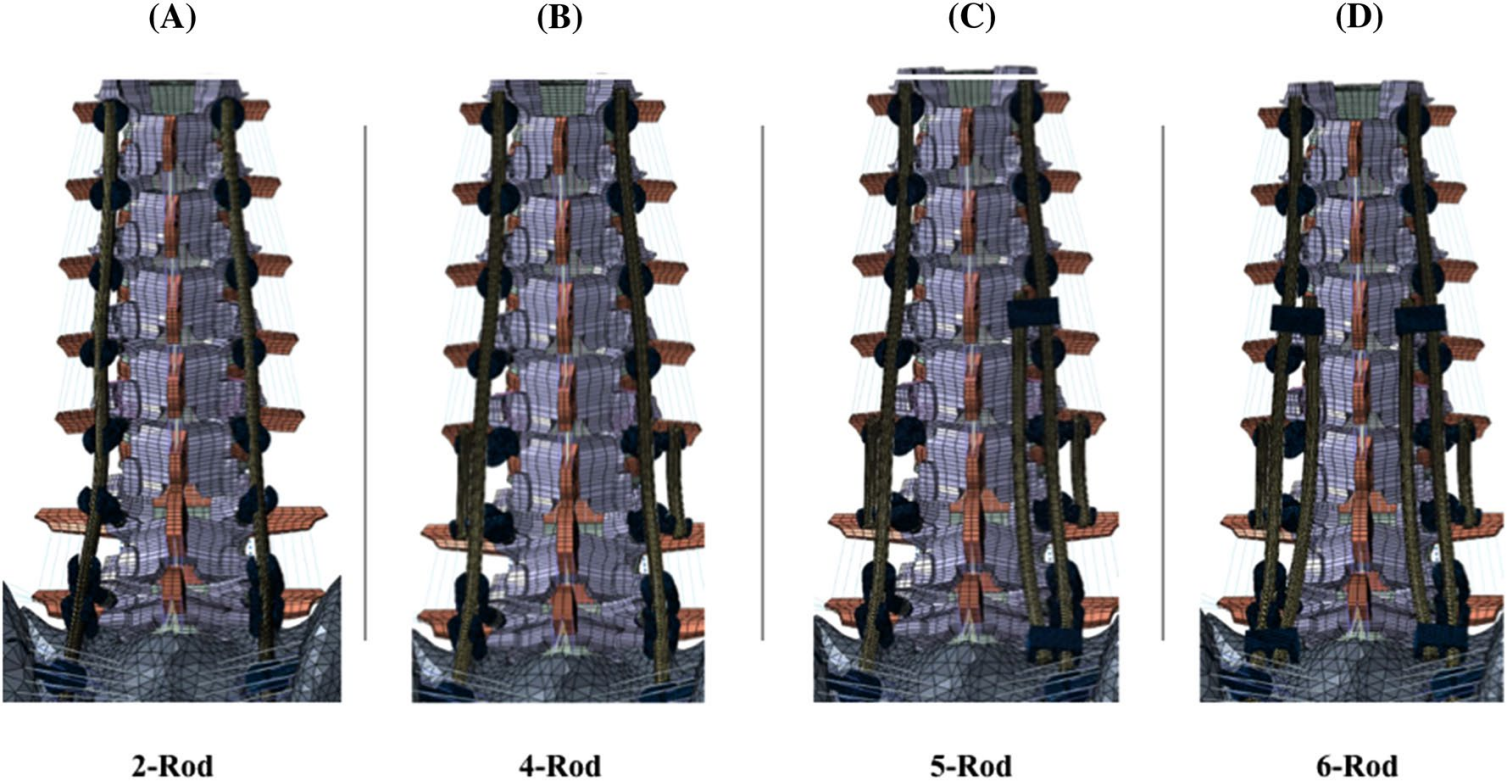
Posterior views of the four various instrumentation configurations used to stabilize lumbar PSO: **a** Two primary rods (Control), **b** 4-rod model with two primary rods and two satellite rods, **c** 5-rod model with two primary rods, two satellite rods, and one accessory rod (attached to the right primary rod with W connectors), **d** 6-rod model with two primary rods, two satellite rods, and two accessory rods (attached to the primary rods with W connectors)

Instrumentation [i.e., rods, pedicle screws, offset/lateral connectors, rod–rod connectors (open up-open up; “W”)] was designed in SolidWorks (Dassault Systems, SolidWorks Corporation, Waltham, MA, USA) software and imported into Abaqus for model development. Each pedicle screw was modeled in two parts (including a tulip and a shaft) connected with a ball and socket joint. The pedicles at T10, T11, and T12 were instrumented with 6.5 × 40 mm poly-axial screws. 6.5 × 45 mm poly-axial screws were used at L1 and L5. Screws with 6.5 mm diameter were chosen at these levels, given prior anatomical studies showing pedicles from T10 to L5 tend to have average pedicle widths > 6.5 mm [20, 21]. Adjacent to the PSO (L2 and L4 levels), 6.5 × 40 mm screws were used so as to allow for the screws to be recessed ventrally relative to the primary rods. The S1 pedicle screws and iliac screws were instrumented with 7.5 × 50 mm and 8.5 × 80 mm poly-axial screws, respectively. A screw length breaching the ventral cortex of S1 was chosen, given a prior biomechanical investigation demonstrating that S1 pedicle screws with tri-cortical fixation (i.e., through the anterior cortex of the sacral promontory) are biomechanically superior to bi-cortical S1 screws not breaching the ventral cortex [22]. The screw sizes were consistent among all four tested configurations. In the “Control” (2-Rods), two 5.5 mm Cobalt–Chromium (CoCr) rods connected all levels from T10 to the pelvis bilaterally. In all other multi-rod configurations (4-rod, 5-rod, 6-rod), these primary rods were connected to all the screws’ tulips from T10 to pelvis, except for L2 and L4 (Fig. 1). Satellite rods spanning the PSO site were used for all multi-rod constructs (outlined below) and were created by securing one on each side to offset/lateral connectors attached to the L2 and L4 pedicle screw tulip heads (Fig. 2).

The following instrumentation configurations were simulated and compared:

- Control (2-rod): Two bilateral rods (one on each side of the spine) from T10-pelvis (Fig. 2A).
- 4-rod: Four-rod configuration consisting of two primary rods combined with two satellite rods spanning the L3 PSO (Fig. 2B).
- 5-rod: Five-rod configuration consisting of two primary rods combined with two satellite rods spanning the L3 PSO as well as one accessory rod connected to the right primary rod via rod–rod connectors between T12 and L1 tulip heads and between the tulip head of S1 and the offset/lateral connector to the iliac bolt (Fig. 2C).
- 6-rod: Six-rod configuration consisting of two primary rods combined with two satellite rods spanning the L3 PSO as well as two accessory rods—each connected to the primary rods via rod–rod connectors between T12 and L1 tulip heads and between the tulip head of S1 and the offset/lateral connector to the iliac bolt (Fig. 2D).

### Finite element model development

The mesh convergence study was performed in two steps. First, a three-point bending simulation was performed on a single screw shaft, and seed sizes were reduced until the differences between the yield loads obtained from two subsequent seed sizes were less than 5%. Similar seed sizes were used in tulip. Second, mesh convergence study was performed on the rods. Seed sizes were reduced until the percentage difference between the forces across the PSO site was below 5%.

To simulate poly-axial screws, two reference points were defined on the screw shaft and the tulip head and the corresponding nodes were coupled to each reference point, separately [23]. Then a Join and Cardan connector was assigned between the shaft and tulip, which constrained the two components in U1, U2, and U3 motions, and allowed for a relative rotation between these components (UR1, UR2, and UR3). Moreover, a surface-to-surface interaction was defined between the tulip and shaft (friction = 0.4 [24]). Rods were tied to tulip and lateral connectors. “W”-connectors were tied to the primary and accessory rods.

A two-step analysis was performed. In step 1, the spine model was pre-loaded with axial compression load to simulate body weight using follower load technique: 300 N to the thoracic spine, 400 N to the lumbar spine, and 400 N to the sacrum [16, 25]. In step 2, pure moments of 7.5 Nm were applied to the top endplate of the T10 vertebra in all three anatomical directions. In both steps, the acetabulum surfaces of the pelvis were fixed in all degrees of freedom. While simulation of axial loading alone is possible, it is not common-place, and was not performed in this study, as instrumentation failure often occurs from complex multi-planar forces.

### Data analysis

For each instrumentation technique, the range of motion (ROM) from T10 to S1 (Global ROM) and between L2 and L4 (PSO ROM) was recorded at the second step (see above) in all directions. The von Mises stresses on the primary, satellite, and accessory rods were also recorded, and percent differences relative to the primary rods in the “Control” (2-rod) model were calculated. The critical von Mises stress locations were recorded and compared between all models. The factor of safety (FOS) was measured to identify the load carrying capacity of the primary rods in each configuration. The FOS describes the strength capacity of a system beyond its expected or actual loads [defined as the ratio of CoCr yield stress (928 MPa) to the maximum von Mises stress observed in primary rods for each configuration (16)]. As such, the greater the FOS, the stronger the material. For each model, the force acting at the PSO site in flexion motion was also captured.

## Results

### T10-S1 range of motion (global ROM) (Fig. 3)

**Fig. 3:**
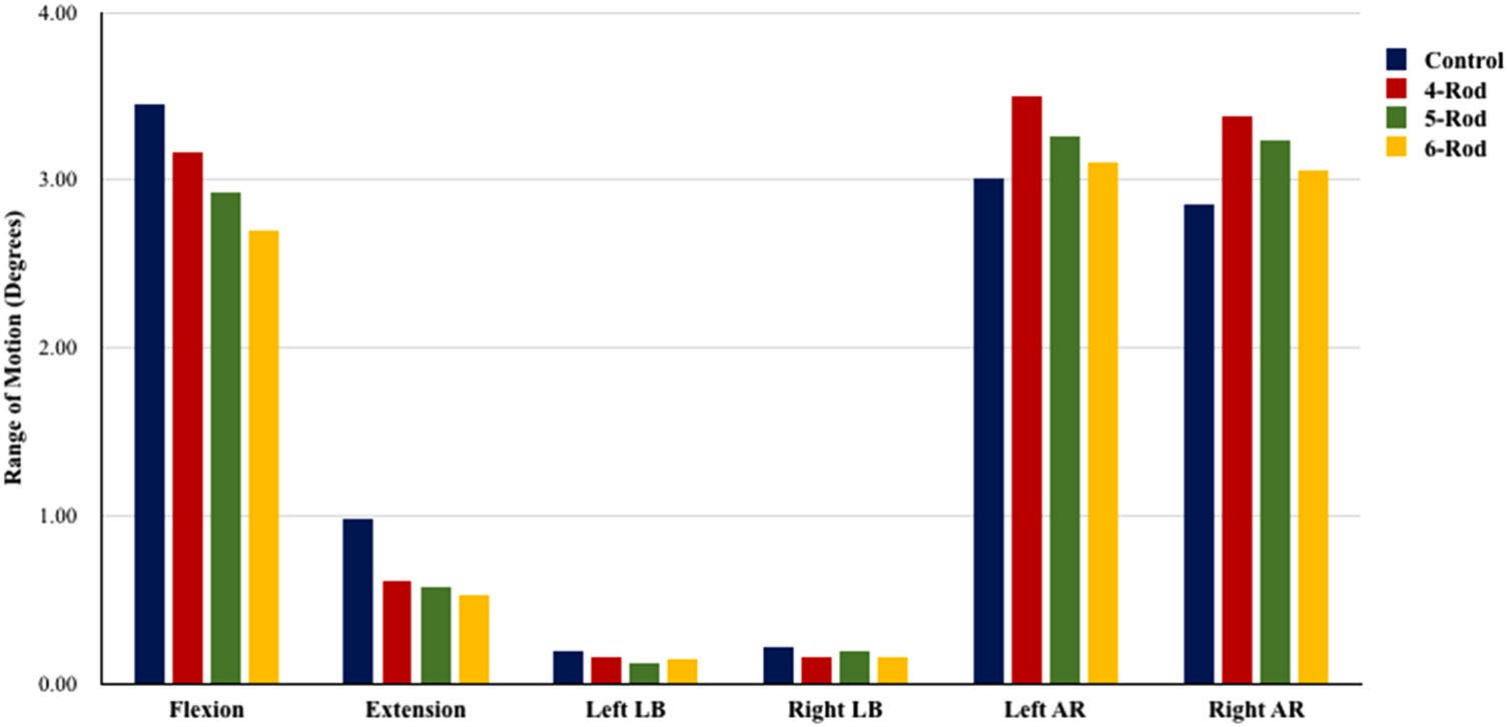
Global (T10-S1) range of motion for 2-rod (Control), 4-rod, 5-rod, and 6-rod configurations

Satellite and accessory rods decreased T10-S1 ROM in flexion–extension and lateral bending while they increased global ROM in axial rotation.

Compared to 2-rods, 4-rods decreased T10-S1 flexion and extension by 8% and 37%, respectively.

5-rod configuration decreased flexion and extension by 15–42% compared to 2-Rods. In the 5-rod constructs, as the accessory rod was connected to the right primary rod, left lateral bending was decreased by 38% while right lateral bending decreased by 9% compared to 2-rods. Moreover, with 5-rods, the left axial rotation increased 8% while right axial rotation increased 13% compared to 2-rods.

6-rods decreased T10-S1 flexion by 22%, extension 47%, and lateral bending by 23–25%, compared to 2-rods. Axial rotation with 6-rods was similar to 2-rods.

### L2–L4 range of motion (PSO ROM) (Fig. 4)

**Fig. 4:**
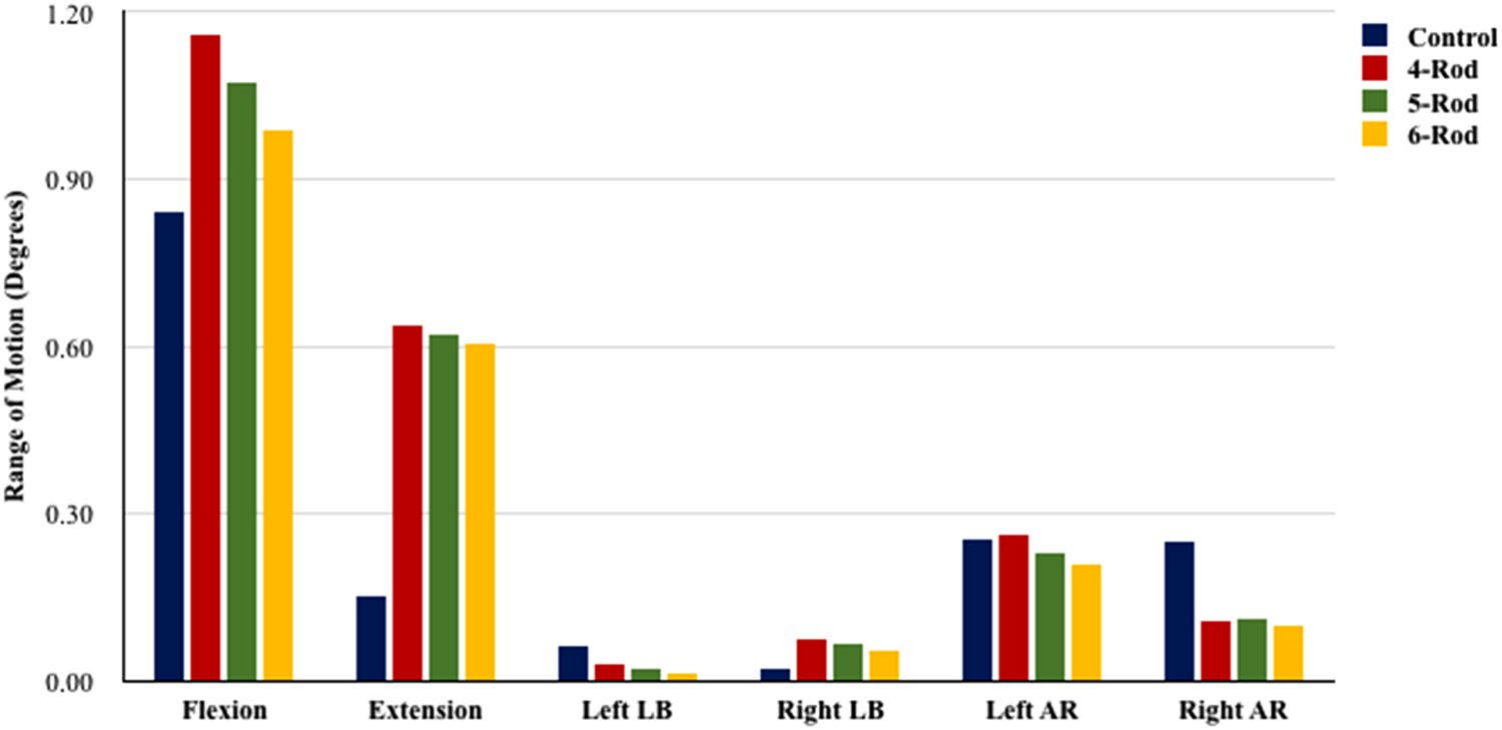
Range of motion at the PSO (L2–L4) for 2-rod (Control), 4-rod, 5-rod, and 6-rod configurations

All multi-rod constructs (4-, 5-, 6-rods) increased L2–L4 ROM compared to 2-rods.

4-rod increased PSO flexion by 38% and extension by 315%, compared to Control (Fig. 4).

Compared to Control, 5-rods increased PSO flexion by 28% and extension by 305%. With 5-rods, left lateral bending at the PSO reduced by 65% while right lateral bending at the PSO increased 205%, compared to Control. 5-rods also reduced left and right axial rotations by 9% and 55%, respectively.

Compared to Control, 6-rods increased L2–L4 flexion by 18% and extension by 295%. With 6-rods, left lateral bending at the PSO was decreased by 78% while right lateral bending at the PSO was increased 152%, compared to Control. Right and left axial rotations were decreased by 18% and 60%, respectively.

As numbers of rods increased, range of motion across the PSO decreased (Fig. 4).

### Rods’ maximum von Mises stresses (Table 2, Fig. 5)

**Table 2:** Values and locations of maximum von Mises stress (MPa) for control and different multi-rod constructs spanning a lumbar PSO

	Rod	Control (2-rod)	4-rod	5-rod	6-rod
Flexion	Primary	294.4 (MPA)	− 30.2%	− 29.9%	− 33.1%
	Satellite	–	− 87.4%	− 89.2%	− 90.5%
	Accessory	–	–	− 43.3%	− 44.9%
	***FOS***	3.2	4.5	4.5	4.7
Extension	Primary	84.4 (MPA)	− 11.13%	− 12.3%	− 23.2%
	Satellite	–	− 70.4%	− 69.5%	− 70.7%
	Accessory	–	–	− 68.2%	− 66.1%
	***FOS***	11.0	12.4	12.5	14.3
Left LB	Primary	216.4 (MPA)	− 24.6%	− 26.5%	− 28.3%
	Satellite	–	− 88.8%	− 88.4%	− 88.87%
	Accessory	–	–	− 45%	− 49.1%
	***FOS***	4.3	5.7	5.8	6.0
Right LB	Primary	217.9 (MPA)	− 29.9%	− 35.8%	− 33.2%
	Satellite	–	− 88.2%	− 87.6%	− 90%
	Accessory	–	–	− 59.5%	− 45.3%
	***FOS***	4.3	6.1	6.6	6.4
Left AR	Primary	258.5 (MPA)	− 19.5%	− 23.3%	− 23.3%
	Satellite	–	− 88.3%	− 87.5%	− 88.5%
	Accessory	–	–	− 43.5%	− 47.9%
	***FOS***	3.6	4.5	4.7	4.7
Right AR	Primary	229 (MPA)	− 7.6%	− 13.5%	− 13.3%
	Satellite	–	− 82.9%	− 82.9%	− 84.8%
	Accessory	–	–	− 55.4%	− 40.1%
	***FOS***	4.1	4.4	4.7	4.7

*Values represent percent differences between stresses on the primary, satellite, and accessory rods relative to the primary rods in the Control model

*FOS* factor of safety

**Fig. 5:**
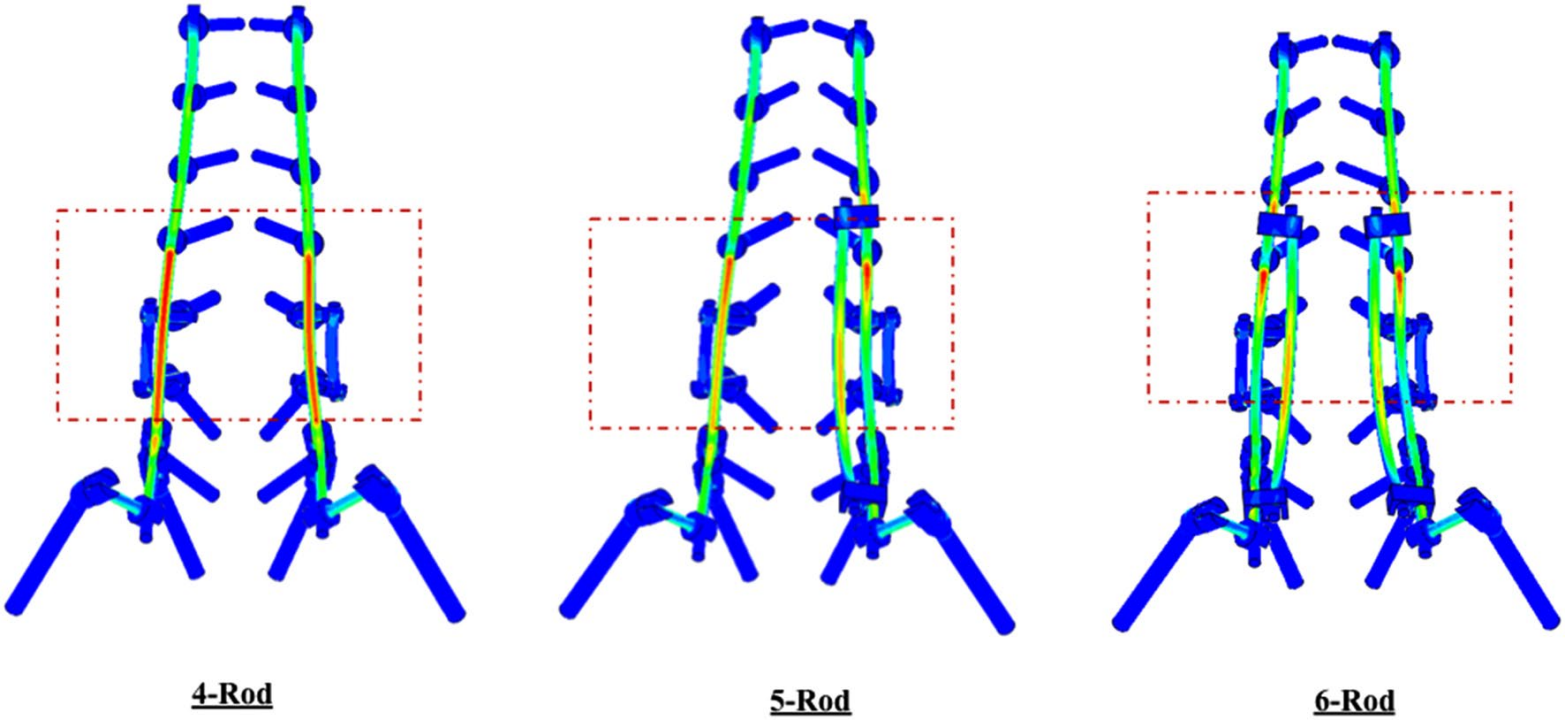
The von Mises stress contour in flexion motion across the posterior instrumentation in the models with 4-rods, 5-rods, and 6-rods

In the 4-rod construct, there was a 7.6–30.2% reduction in the primary rods’ von Mises stresses and a 70.4–88.8% reduction on satellite rods’ stresses, compared to 2-rods.

Compared to 2-rods, 5-rods showed 12.3–35.8% reduction in the primary rods’ von Mises stresses. With 5-rods, satellite rods showed 69.5–89.2% lower von Mises stresses than the Control’s primary rods. Additionally, the accessory rod as part of the 5-rod configuration showed 43.3–68.2% lower von Mises stresses than the Control’s primary rods.

In the 6-rod construct, primary rod stresses decreased 13.3–33.2%. With 6-rods, von Mises stresses on satellite rods were 70.7–90.5% lower than the Control’s primary rods. Moreover, accessory rods experienced 40.1–66.1% lower stresses than the Control’s primary rods.

Considering the maximum recorded von Mises stresses on the primary rods, the 6-rod configuration showed the highest factor of safety (FOS), followed by the 5-rod technique in all motions except right lateral bending (Table 2). The 2-rod configuration had the lowest FOS in all motions (Table 2).

Relative to 4-rods, addition of one accessory rod did not have a significant effect on the primary rods’ stresses. However, the addition of two accessory rods (6-rod) led to a higher reduction in the primary rods’ stresses. Moreover, one or two accessory rods did result in a higher reduction in the satellite rods’ stresses (Table 2, Fig. 5).

In 4-rods, two critical stress locations were observed: adjacent to the PSO site and at the L5-S1 level. The critical stress location was observed on primary rods for all constructs. Adding the accessory rods to the satellite rods shifted the critical stress locations away from the area between the apex of the primary rods and position of W connectors in some motions (Fig. 5).

### Forces at the PSO

Compared to the model with 2-rods (336 N), 4-rods (348.6 N) and 5-rods (343.2) increased the force across the PSO. However, the 6-rod configuration demonstrated a lower forces across the osteotomy site (324.2 N) compared to Control (336 N).

## Discussion

The ideal construct to stabilize a lumbar PSO is an area of greatest interest and one that continues to evolve. While 4-rod constructs have consistently demonstrated decreased rates of rod fracture compared to 2-rods, non-unions and rod fractures still occur in the setting of 4-rods stabilizing lumbar PSOs. To further minimize the risk of rod fractures, “super” multi-rod constructs (5-, 6-rods) across lumbar PSOs have been implemented; however, little is known about these constructs’ biomechanics. As such, this study evaluated the biomechanical characteristics of increasing number of rods (4-, 5-, 6-rod) across a lumbar PSO. The major findings were that all multi-rod constructs, relative to 2-rods, decreased global ROM, decreased PSO ROM, decreased von Mises stresses on the primary rods, and increased FOS for the primary rods. Additionally, with increasing number of rods (4- to 5- to 6-), ROM across the PSO decreased (increased rigidity), leading to decreased forces across the anterior column (PSO), decreased von Mises stresses on the primary rods, and increased FOS of the primary rods. While the results of this study are concordant with prior biomechanical evaluations, they do provide unique information on the biomechanical effects of 5-rod and 6-rod constructs across lumbar PSOs.

Relative to 2-rods, 4-rods spanning a lumbar PSO have consistently been found to decrease global ROM, decrease ROM and forces at the PSO site, and decrease rod stresses across the PSO site [8–11, 13, 18, 27]. This has been mirrored by clinical studies that demonstrate 4-rod constructs result in lower rates of non-unions and rod breakage at the PSO site [7, 14, 23]. For example, Gupta et al*.* reported for lumbar PSO a rod breakage rate of 25% with 2-rods and 0% with 4-rods (*p* = 0.008), and a pseudarthrosis rate of 25% with 2-rods and 3.4% with 4-rods (*p* = 0.035) [6]. Additionally, Hyun et al*.* showed 29% of patients with 2-rod constructs experienced pseudarthrosis, while patients treated with 4-rod constructs had a significantly lower rate (15%) [7]. Moreover, in patients with 4-rods, 80% of the pseudarthroses occurred above and/or below the osteotomy site while 20% happened at the PSO level [7].

Four-rod constructs across a lumbar PSO can be achieved in a variety of ways using satellite rods (rods not connected to the main rods) and/or accessory rods [15]. Attempts have been made to elucidate biomechanically the ideal configuration of 4-rod constructs. Data from Vosoughi et al. suggested that there was significant benefit in supplementing medial over lateral accessory rods across a lumbar PSO [16]. It was also reported that short, recessed, in-line satellite rods across a lumbar PSO offered the best biomechanical environment, as it was the only construct to increase PSO forces while all the 4-rod constructs created with accessory rods (no satellite rods) decreased the magnitude of the load acting across the osteotomy region [16]. More recently, our group found that a 4-rod construct created with satellite rods connected to lateral connectors above/below the PSO site provides an even more favorable biomechanical environment compared to the aforementioned 4-rod technique with in-line, recessed satellite rods, as it increased PSO ROM, increased force magnitude across the PSO site, and demonstrated less stress shielding of the posterior instrumentation (unpublished data). In turn, we chose to use this 4-rod configuration with lateral satellite rods across the PSO as a point of reference to which the 5- and 6-rod constructs were compared in this study.

The biomechanical effects of creating 5-rod and 6-rod constructs using a combination of satellite rods and accessory rods are of interest. First to note is that 5- and 6-rods increased the construct’s global rigidity and led to lower global ROMs compared to 2-rod and 4-rod configurations. In the 5- and 6-rod techniques, the posterior load was distributed across five or six components, resulting in a reduction in von Mises stresses in each component [13]. In models with additional accessory rods (5- and 6-rod), we also noted that locations of maximum von Mises stresses were shifted to the areas between the PSO site and rod–rod (“W”) connectors in several motions, likely due to the stress concentration produced by the W connectors. This is different than 4-rods where the critical stress locations are traditionally observed either adjacent to the PSO site or at the L5-S1 level [2].

Compared to 4-rods, 5-rods have slightly greater PSO ROM, slightly lower von Mises stresses on the primary rods in all motions except for flexion, and slightly higher FOS of the primary rods in lateral bending and axial rotation. When evaluating 6-rods, compared to 4-rods, it is clearer that 6-rods provide more notable reductions in PSO ROM, lower von Mises stress on the primary rods in all motions, and higher FOS of the primary rods in all motions. These data suggest that 6-rods provide the most rigid environment and stress shielding of the posterior instrumentation. While this may be considered favorable because the rods’ stresses are the lowest, it comes at the expense of offloading the anterior column. Note that compared to 4-rod, 5-rod resulted in a 5.4 N lower PSO force (348.6 N vs. 343.2 N), while 6-rod produced a 24.4 N lower PSO force compared to 4-Rods (348.6 N vs. 324.2 N). What is more striking is that the PSO force in 6-rods (324.2 N) was lower than 2-rods/Control (336 N), whereas the PSO forces in the setting of 4-rod (348.6 N) and 5-rod (343.2 N) were greater than 2-rods (336 N). As a higher PSO force is postulated to promote bone-healing and fusion at the osteotomy site anteriorly and decrease the chances of non-union [17], the 5-rod configuration may represent the happy medium between reduction of stresses on the primary rods and maintenance of adequate PSO forces for anterior healing. Conversely, 4-rods may not produce a rigid enough construct to adequately protect the posterior instrumentation while 6-rods produce such a rigid a construct posteriorly that it jeopardizes the anterior column’s ability to heal.

The results of this study should be considered in the context of its limitations. While we believe the accuracy of this FEA model is acceptable given its use of a well-established, previously validated model of a L3 PSO, there are several factors that may jeopardize its accuracy in simulating the forces during a PSO. These include simulation performed with no muscle forces, lack of range of motion data for the cadaveric spine with lateral satellite rod configuration and using uncomplicated geometries of the implants and simplified contact and constraints. Moreover, the residual stresses produced as a result of rod contouring and screw/rod tightening were not considered. Specifically, the interconnections of the screws, rods, lateral connectors, and anatomy were all in ideal conditions, which is almost never the case clinically. Additionally, satellite and accessory rods’ effects on biomechanics may be influenced by other factors, including rod characteristics (i.e., diameter, material, bend magnitude). However, it should be noted that while the model has these limitations, the use of comparative analyses (relative to the Control/2-rod Configuration) makes our reported relative differences of greater credence than individual absolute values. While we report relative differences between the different rod configurations, we are unable to comment upon the biomechanical and clinical significances of our observed biomechanical differences and relative long-term clinical performance of the different instrumentation configurations evaluated in this study, particularly because the exact margin of error as well as the margin of important difference is not known and because the utilization of laterally based satellite rods as well as 5-rod and 6-rod constructs is relatively new for PSO closure and stabilization. Furthermore, although the use of more rods is intended to decrease the risk of developing a pseudarthrosis, the addition of more rods also theoretically may interfere with the development and/or the maturation of a fusion mass. While understanding of how much of an adverse effect additional instrumentation has in jeopardizing bony deposition is needed, investigating this is beyond the scope of this study, as it cannot be evaluated by finite element analysis. Future investigations should ideally aim to address these important questions. Other comparisons that were beyond the scope of this study, but would also be important avenues for future investigations, were determination of the relative biomechanics between different PSO levels (L2 vs. L3 vs. L4 vs. L5 vs. S1) and the biomechanical effects on “super” multi-rod constructs with anterior column support adjacent to the PSO site. We used L3 as the osteotomy site, given the majority of prior biomechanical studies have used L3 as the PSO site [8, 10–13, 16]; however, it is important to note that level of osteotomy is important for restoring the appropriate lumbar shape and lordosis based on Roussouly types [26, 27]. As such, L4 or L5 PSO may be more appropriate for patients without high pelvic incidences. Furthermore, prior investigations have demonstrated that anterior column support adjacent to a lumbar PSO decreases posterior rod strains, increases dynamic stiffness and fatigue bending, and decreases axial rotation [9, 12]. As our evaluation did not include anterior column support adjacent to the PSO, we cannot comment upon the effect of interbody support on the biomechanics of “super” multi-rod constructs. Despite these limitations, this is the first study to report on the relative biomechanics of “super” multi-rod (5- and 6-rod) constructs and will hopefully stimulate further discussion and inquiry into their clinical utility.

## Conclusion

In this FE analysis of an L3 PSO stabilized with different multi-rod constructs, 4-rods across the PSO reduced stresses on primary rods. Adding accessory rods (5-rod and 6-rods total) increased the construct’s rigidity and led to a lower global ROM and PSO ROM. Moreover, 5- and 6-rods resulted in forces on the posterior instrumentation being distributed across more components, which decreased force at the PSO site compared to 4-rod and 2-rods. Although increased rigidity afforded by 5- and 6-rods decreased rod stresses, it should be highlighted that due to higher stiffness, less load was transferred to the anterior vertebral column (particularly for 6-rod), which may not be favorable for healing of the anterior column. Based on this FE analysis, a balance between the construct’s rigidity and anterior load sharing is essential.

## Supplementary Information

Below is the link to the electronic supplementary material.

Supplementary file1 (JPEG 88 KB)

Supplementary file2 (JPEG 358 KB)

## Data Availability

The data used to support the findings of this study are available from the corresponding author upon request.
