# Supplementary figures and images for: Does number of rods matter? 4-, 5-, and 6-rods across a lumbar pedicle subtraction osteotomy: a finite element analysis
Source: Spine Deform. 2022 Dec 9;11(3):535–43. doi: 10.1007/s43390-022-00627-0 (PMC10147790; doi:10.1007/s43390-022-00627-0)

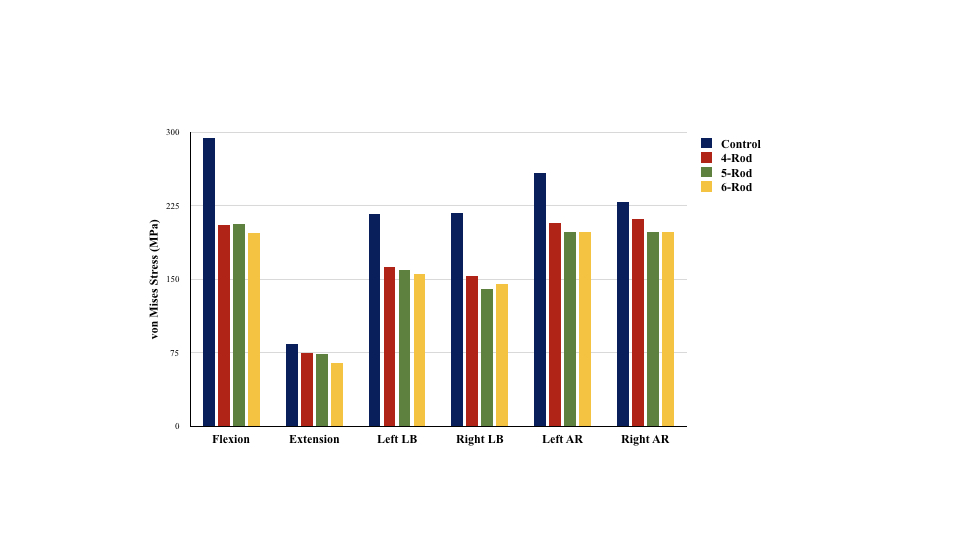

Supplement: Supplementary file 1 — Supplementary file1 (JPEG 88 KB) [file 43390_2022_627_MOESM1_ESM.jpeg]

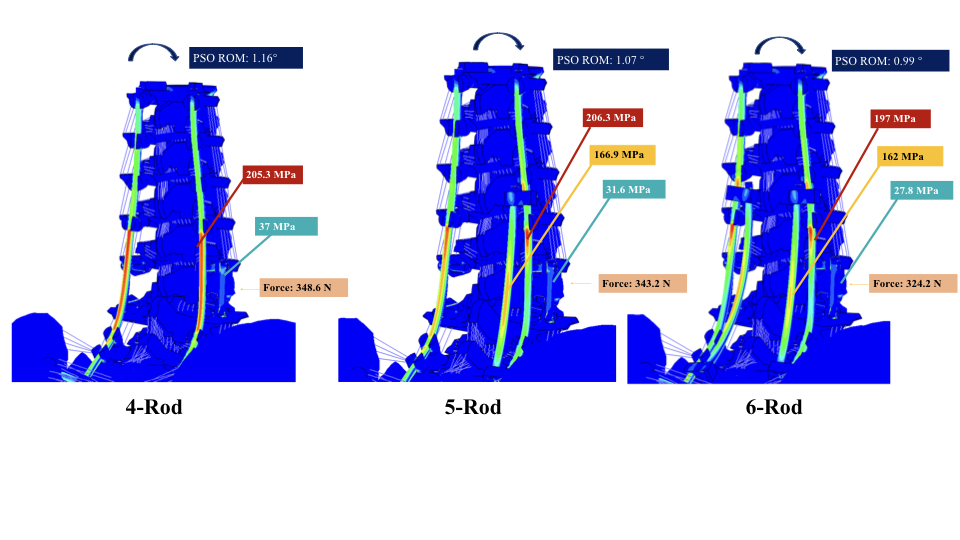

Supplement: Supplementary file 2 — Supplementary file2 (JPEG 358 KB) [file 43390_2022_627_MOESM2_ESM.jpeg]
